# ESKALE study, a French real-world study of esketamine nasal spray for patients with treatment-resistant depression

**DOI:** 10.1192/j.eurpsy.2022.693

**Published:** 2022-09-01

**Authors:** S. Ludovic, M.A. Codet, M. Rotharmel, P. De Maricourt, J. Boursicot, E. Gaudre Wattinne

**Affiliations:** 1 CHU Clermont-Ferrand, Department Of Psychiatry, University Of Auvergne,, Clermont-Ferrand, France; 2 Janssen Cilag, Medical Affairs, Issy les Moulineaux, France; 3 CH Rouvray, Department Of Psychiatry, Sotteville-lès-Rouen, France; 4 CH Ste Anne, Department Of Psychiatry, Paris, France

**Keywords:** esketamine, Treatment Resistant Depression, retrospective study, Real World Evidence

## Abstract

**Introduction:**

Esketamine nasal spray has been developed to treat adults with treatment resistant depression. On Dec.2019, EMA granted a market access approval in this indication.

**Objectives:**

ESKALE is a descriptive study of treatment resistant depression patients treated with esketamine in France.

**Methods:**

Observational retrospective study. 157 patients are included in 3 cohorts depending on their treatment initiation date. This abstract presents the second interim results of patients treated with esketamine and whom data collection ranges from Oct.2019 and Sept.2021.

**Results:**

66.7% of patients were females. Average age was 49 years old with 26 patients > 65 years old. Duration of the current depressive episode was 26.0 months (mean). 48.8% of patient have > 1 suicide attempt during whole life. At esketamine initiation, 78.2% patients were clinically perceived to have severe depression with a MADRS score of 32.4 (median) and a PHQ9 score of 19.5 (median). For the overall sample, esketamine was prescribed in median as a 3^rd^ line and for 40.5% of patients after neurostimulation. The majority of the patient started esketamine at 28 mg or 56 mg and increased the dose to 84 mg. After 4 months of treatment, clinical benefits are the following: decrease of MADRS total score -16.5 points (median) corresponding to 58% of responders and a PHQ9 total score decrease of -8.6 points (median). No new safety signal detected.

**Conclusions:**

This second interim analysis describes patients’ profiles and clinical evolution over a longer period and a broader population than the first interim analysis. The conditions of use are consistent with the ones approved by health authorities.

**Disclosure:**

I (Marie-Alix Codet) works as a full employee at Janssen Cilag

